# Multi-Modal Machine Learning for Evaluating the Predictive Value of Pelvimetric Measurements (Pelvimetry) for Anastomotic Leakage After Restorative Low Anterior Resection

**DOI:** 10.3390/cancers17061051

**Published:** 2025-03-20

**Authors:** Ritch T. J. Geitenbeek, Simon C. Baltus, Mark Broekman, Sander N. Barendsen, Maike C. Frieben, Ilias Asaggau, Elina Thibeau-Sutre, Jelmer M. Wolterink, Matthijs C. Vermeulen, Can O. Tan, Ivo A. M. J. Broeders, Esther C. J. Consten

**Affiliations:** 1 Department of Surgery, Groningen University Medical Center, University of Groningen, 9713 GZ Groningen, The Netherlands; r.t.j.geitenbeek@umcg.nl (R.T.J.G.); m.broekman@umcg.nl (M.B.); frieben@stud.uni-heidelberg.de (M.C.F.); 2 Department of Surgery, Meander Medical Center, 3813 TZ Amersfoort, The Netherlands; sc.baltus@meandermc.nl (S.C.B.); sn.barendsen@meandermc.nl (S.N.B.); i.asaggau@students.uu.nl (I.A.); mvermeulen2@diakhuis.nl (M.C.V.); iamj.broeders@meandermc.nl (I.A.M.J.B.); 3 Department of Robotics and Mechatronics, University of Twente, 7522 NB Enschede, The Netherlands; c.o.tan@utwente.nl; 4 Department of Surgery, University of Heidelberg, 69117 Heidelberg, Germany; 5 Department of Applied Mathematics, Technical Medical Center, University of Twente, 7522 NB Enschede, The Netherlands; e.thibeau-sutre@utwente.nl (E.T.-S.); j.m.wolterink@utwente.nl (J.M.W.)

**Keywords:** colorectal cancer, low anterior resection, anastomotic leakage, pelvimetry, machine learning

## Abstract

**Background/Objectives**: Anastomotic leakage (AL) remains a major complication after restorative rectal cancer surgery, with accurate preoperative risk stratification posing a significant challenge. Pelvic measurements derived from magnetic resonance imaging (MRI) have been proposed as potential predictors of AL, but their clinical utility remains uncertain. **Methods**: This retrospective, multicenter cohort study analyzed rectal cancer patients undergoing restorative surgery between 2013 and 2021. Pelvic dimensions were assessed using MRI-based pelvimetry. Univariate and multivariate regression analyses identified independent risk factors for AL. Subsequently, machine Learning (ML) models—logistic regression, random forest classifier, and XGBoost—were developed to predict AL using preoperative clinical data alone and in combination with pelvimetry. Model performance was evaluated using F1 scores, with the area under the receiver operating characteristic (ROC-AUC) and precision–recall curves (AUC-PR) as primary metrics. **Results**: Among 487 patients, the overall AL rate was 14%. Multivariate regression analysis identified distance to the anorectal junction, pelvic inlet width, and interspinous distance as independent risk factors for AL (*p* < 0.05). The logistic regression model incorporating pelvimetry achieved the highest predictive performance, with a mean ROC-AUC of 0.70 ± 0.09 and AUC-PR of 0.32 ± 0.10. Although predictive models that included pelvic measurements demonstrated higher ROC-AUCs compared to those without pelvimetry, the improvement was not statistically significant. **Conclusions**: Pelvic dimensions, specifically pelvic inlet and interspinous distance, were independently associated with an increased risk of AL. While ML models incorporating pelvimetry showed only moderate predictive performance, these measurements should be considered in developing clinical prediction tools for AL to enhance preoperative risk stratification.

## Simple Summary

Anastomotic leakage is a serious complication following rectal cancer surgery, which can lead to poor outcomes and prolonged recovery. The accurate preoperative identification of patients at higher risk is challenging. In this study, we analyzed pelvis measurements obtained from MRI scans, known as pelvimetry, to assess whether these dimensions could help predict the likelihood of anastomotic leakage. We also used machine learning models to combine pelvimetry with clinical data and evaluate their predictive performance. Our results identified key pelvic dimensions, such as the pelvic inlet width and interspinous distance, as potential risk factors for anastomotic leakage. While the predictive models showed moderate performance, these findings suggest that incorporating pelvimetric measurements into clinical risk assessments may help surgeons improve preoperative planning and patient safety in rectal cancer surgery.

## 1. Introduction

Rectal carcinoma, a malignancy originating from the rectal mucosa, represents a significant portion of colorectal cancers and poses a considerable clinical challenge due to its anatomical location and potential for local invasion. The standard treatment for locally advanced rectal carcinoma is total mesorectal excision (TME) [1]. This surgical procedure involves the precise removal of the rectum and the surrounding mesorectal tissue to achieve oncological clearance. While TME has markedly improved local control and survival rates, the creation of an anastomosis—where the surgeon reconnects the bowel after tumor resection—carries the risk of anastomotic leakage (AL), a complication associated with substantial morbidity, increased mortality, and prolonged hospital stays [2].

AL following rectal surgery is a critical issue, with reported rates varying from 4% to 20% depending on patient demographics, tumor characteristics, and surgical techniques [3]. This complication can lead to severe consequences such as sepsis, reoperation, permanent stoma formation, and even death. Identifying patients at higher risk for this complication is essential for guiding surgical decision-making, improving outcomes, and potentially preventing leaks. Previous studies have identified several risk factors for AL, including male sex, obesity, tumor location, preoperative radiotherapy, and technical aspects of the surgery [3,4,5,6]. However, the predictive value of these factors remains limited, often leading to inconsistent clinical application.

Repeatedly, pelvic anatomical features have been discussed with regard to their potential impact on the difficulty of TME as well as the associated risk of AL. A narrow and deep pelvis is thought to increase the technical difficulty of TME, which may contribute to higher rates of AL [7,8,9,10,11,12]. Despite these theoretical implications, pelvimetric measurements—used to assess pelvic dimensions—have not been thoroughly evaluated in predicting AL. This gap in understanding limits current risk stratification in rectal cancer surgery.

Machine learning (ML) facilitates the objective analysis of complex medical data, enabling advanced applications in preoperative risk prediction [13]. In the context of colorectal surgery, ML has been employed to predict surgical outcomes with promising results [14,15]. Several studies have developed ML models based on electronic medical record data, but studies incorporating pelvic measurements in the ML prediction are scarce [16].

To address this gap, the present study aims to evaluate the predictive value of patient characteristics, tumor characteristics, and pelvic measurements for AL in a large cohort of rectal carcinoma patients. By integrating patient and tumor characteristics with pelvimetry in an ML model, we aim to provide a robust preoperative prediction model for AL that enhances patient outcomes and refines surgical strategies for treating rectal carcinoma. Model performance will be assessed using established metrics, including the F1 score, area under the precision–recall curve (AUC-PR), area under the receiver operating characteristic curve (AUC-ROC), sensitivity, and specificity, ensuring a comprehensive evaluation of predictive accuracy.

## 2. Materials and Methods

### 2.1. Study Design and Population

This retrospective, multicenter cohort study was conducted under the Minimally Invasive Rectal Cancer Taskforce (MIRECA) to evaluate the predictive value of pelvic measurements for AL following restorative low anterior resection (RLAR). The MIRECA dataset has been detailed previously [17]. All patients who underwent rectal resection for MRI-defined primary rectal adenocarcinoma between 1 January 2013, and 31 December 2021, were identified from the national Dutch ColoRectal Audit (DCRA) database. This database is a comprehensive, prospective, and obligatory nationwide registry with independent oversight, ensuring the accuracy and reliability of the data.

The study involved eleven dedicated high-volume centers, each performing at least 40 TME procedures annually, a criterion ensuring surgical expertise and standardization of care across sites [18,19]. A local multidisciplinary cancer board reviewed all patients, and (neo)adjuvant therapy was administered following the Dutch National Guidelines [20]. Postoperative follow-up was performed according to these national guidelines.

### 2.2. Inclusion and Exclusion Criteria

Patients were included in the study if they met the following criteria: (1) diagnosis of MRI-defined primary rectal adenocarcinoma based on the diagnosis of MRI-defined primary rectal adenocarcinoma within 15 cm from the anorectal junction (ARJ), (2) underwent robotic or laparoscopic LAR with curative intent, and (3) had a preoperative pelvic MRI available that included visualization of the entire bony pelvis extending to the sacral promontory for comprehensive pelvimetric measurements.

Exclusion criteria were stringently applied to maintain cohort uniformity. Patients were excluded if they (1) underwent minimally invasive TME at a center that had not yet fulfilled the learning curve requirements, (2) underwent local excision alone, (3) underwent transanal endoscopic microsurgery (TEM) prior to TME, (4) underwent non-restorative low anterior resection (NRLAR), (5) underwent abdominoperineal resection (APR), (6) presented with metastatic disease (cM1), (7) had artifacts on the MRI scans or a field of view that did not include the entire bony pelvis from the coccyx to the sacral promontory, or (8) were operated on in an emergency setting.

### 2.3. Ethical Considerations

The study followed the Strengthening the Reporting of Observational Studies in Epidemiology (STROBE) guidelines to ensure transparency and completeness in the reporting of observational research [21]. The Medical Research Ethics Committees United (MEC-U) (AW19-023/W18.100) reviewed the study protocol and waived the need for informed consent, as all data were anonymized. Ethical approval was also obtained from the local ethics boards of all participating institutions, ensuring compliance with national and international ethical standards for clinical research.

### 2.4. Outcomes and Definitions

The primary outcome of this study was AL, defined as a disruption of gastrointestinal continuity at the anastomosis site. Indicators of AL included a pelvic abscess adjacent to the anastomosis or peritonitis. A pelvic abscess was characterized as a radiologically confirmed collection in the pelvic cavity that required antibiotic therapy. This study encompassed all forms of AL, including biochemical, radiological, and clinical types, classifying them using the International Study Group of Rectal Cancer (ISREC) criteria as follows: grade A (subclinical, managed with observation or medication), grade B (requiring radiological or transanal drainage), and grade C (requiring re-laparotomy) [22]. AL incidence was monitored until 3 years after TME surgery. Demographic and clinicopathological variables were defined based on standardized criteria. The distance to the ARJ was measured from the tumor’s proximal border to the ARJ using MRI.

### 2.5. MRI Pelvimetry

**Figure 1:**
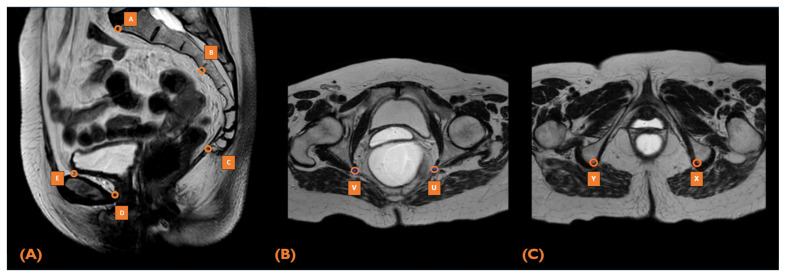
A visualization of MRI acquisition with the pelvimetry landmarks. (**A**) Sagittal MRI with the promontorium (A), S3-vertebrae (B), coccyx (C), dorsal part of os pubis (D), cranial part of os pubis (E). (**B**) Transversal MRI with the left and right ischial tuberosities (U and V). (**C**) Transversal MRI with the left and right ischial spines (X and Y). Abbreviations: MRI, magnetic resonance imaging.

All included rectal cancer patients underwent preoperative MRI examination for tumor staging. Pelvic dimensions were measured from mid-sagittal, axial, and transverse pelvic sections using concatenated T2-weighted sagittal Turbo-spin-echo sequences from the standardized abdominal pelvic MR imaging protocols using 1.5- and 3.0-Tesla (T) MRI scanners (Siemens Medical Solutions, Forchheim, Germany; Philips Medical Systems, Best, The Netherlands). Pelvimetry was performed using predefined landmarks and measurements grounded in the existing literature and consensus from senior multidisciplinary co-authors. The pelvic dimension measurements were obtained based on nine anatomical landmarks visualized on sagittal and transversal MRI volumes (Figure 1). The landmarks comprised the promontory (A), S3-vertebrae (B), coccyx (C), dorsal part of the os pubis (D), cranial part of the os pubis (E), lowest points of the left and right ischial tuberosities (U and V), and the tips of the left and right ischial spines (X and Y). Two trained medical doctors (MDs) independently conducted the pelvimetric measurements, following specialized training in colorectal imaging under the guidance of a dedicated abdominal radiologist. Both MDs were blinded to patient outcomes, and protocol adherence was validated by a senior radiologist, who audited 20% of the cases. The senior radiologist adjudicated discrepancies, particularly those exceeding 5% interobserver differences. All measurements were based on preoperative abdominal MR images, with the most recent MRI before surgery used in cases where neoadjuvant therapy had been administered.

A narrow, deep pelvis has been described to influence the surgical difficulty of TME. Therefore, the following pelvimetry measurements were used as input for the machine-learning model [23]: 1. Sacrococcygeal distance: length between promontory (A) and coccyx (C); 2. Pelvic inlet: length between promontory (A) and cranial part of os pubis (E); 3. Pelvic outlet: length between coccyx (C) and dorsal part of os pubis (D); 4. Pelvic depth: length between coccyx (C) and the midpoint (F) between promontory (A) and cranial part of os pubis (E); 5. Intertuberous distance: length between right ischial tuberosity (U) and left ischial tuberosity (V); 6. Interspinous distance: narrowest span between the right ischial spine (X) and left ischial spine (Y); 7. Sacral angulation: angle between promontory (A), S3 vertebrae, and coccyx (C); 8. Pelvic area: approximation of the lesser pelvis surface between the five sagittal landmarks (A–E); and 9. Pelvic volume: approximation of the volume by calculating the frustum-shaped cone based on the pelvic inlet and outlet lengths and height differences.

### 2.6. Statistical Analysis

Statistical analysis was performed using Python 3.10.2 (Python Software Foundation). Missing data were handled using multiple imputations by chained equations (MICE), assuming data were missing at random (MAR). For variables not included in the predictive models, missing values did not exceed 15% of the dataset. Percentages reported in this study represent the available data, excluding cases with missing information. Categorical variables are presented as absolute numbers and percentages, while continuous variables are expressed as mean ± standard deviation (SD) or median with interquartile range (IQR), depending on the distribution.

### 2.7. Assessment of Predictive Value

First, the significance and strength of the relationship between the preoperative variables (clinical characteristics and pelvimetry) and AL were assessed. The Shapiro–Wilk test was performed to assess the normality of variables. Depending on the distribution, the Point-Biserial Correlation or the Spearman correlation was used to assess the univariate relationship for continuous variables. Binary and categorical variables were analyzed using the chi-squared test, with Cramer’s V used to measure their univariate relationship. Confidence intervals for correlation coefficients were calculated using Fisher’s z-transformation. Statistical significance was set at a *p*-value of less than 0.05. Multivariate regression analysis was performed by backward selection of the significant variables in the univariate analysis. The clinical variables were selected based on established risk factors documented in the literature. The following factors were analyzed: age at surgery, gender (male), height (cm), weight (kg), body mass index (BMI), American Society of Anesthesiologists (ASA) score, history of abdominal surgery, clinical TNM-stage, the distance of tumor to the ARJ, neoadjuvant therapy (none, short-course radiotherapy, long-course radiotherapy, and chemoradiation), surgical approach (laparoscopic/robotic), and pelvimetric measurements.

**Figure A1:**
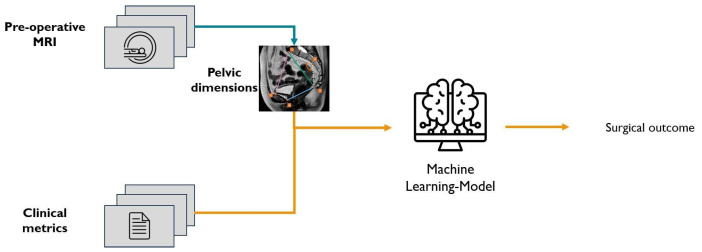
An outline of the multi-modal preoperative prediction of TME outcomes. Critical pelvic dimensions are extracted from the preoperative MRI, which is routinely acquired for tumor staging. The dimensions and clinical metrics are combined in a machine-learning model to assess the surgical outcome comprehensively. Abbreviations: MRI, magnetic resonance imaging.

Second, an ML model for the preoperative prediction of AL was developed based on clinical and pelvimetry data. An outline of the pipeline for handling the multi-modal data is shown in Appendix A Figure A1. Three ML models were assessed: logistic regression (LR), random forest classifier (RFC), and XGBoost (XGB). Multiple analyses were applied to each model type. First, Lasso feature regression analysis was applied to select the most relevant preoperative variables and minimize multicollinearity. Second, a nested, stratified five-fold cross-validation was applied. For each fold, 80% of the data was used to train the model, while the remaining 20% was reserved for testing. The validation based on five distinct outer splits enabled the assessment of the model’s performance on all patients. The five inner splits enabled hyperparameter tuning. The stratification ensured consistent data distribution between positive and negative surgical outcomes. Bayesian hyperparameter tuning was performed based on the F1 score. The tuning of the parameters depended on the model type, and the data imbalance was compensated for by either class weighting or SMOTE (Synthetic Minority Oversampling Technique). We evaluated the class weighting ratios of 1:1, 1:5, 1:7, and 1:9 or used the standard and borderline SMOTE for upsampling of the minority class (anastomotic leakage) based on the five nearest neighbors. Finally, the best-performing model was chosen based on the F1 score. Model development was performed based on only preoperative clinical parameters or both clinical parameters and pelvimetry to assess its additional value. The performance of the models was evaluated through the F1 score, the AUC-PR, the AUC-ROC, sensitivity, and specificity.

## 3. Results

### 3.1. Patient Characteristics

**Figure 2:**
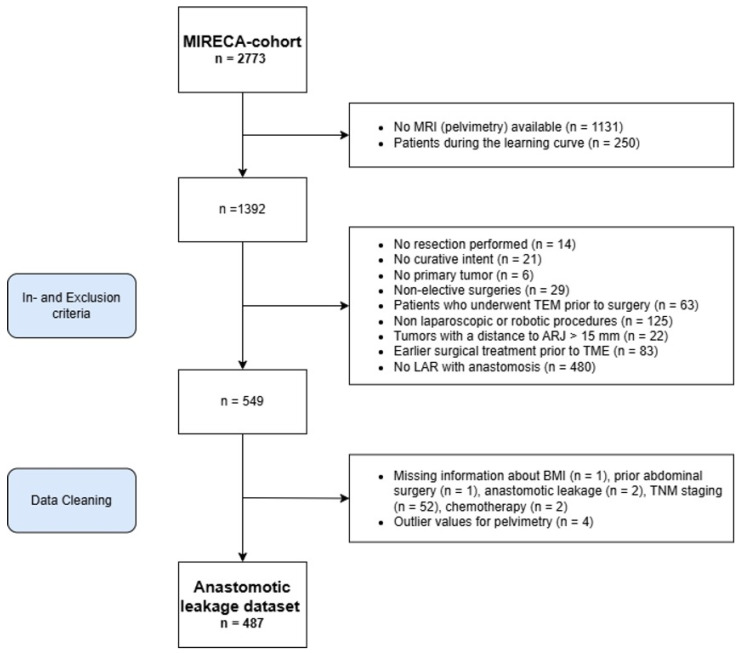
Flowchart of patient selection for machine learning model development. Abbreviations: ARJ, anorectal junction; BMI, body mass index; LAR, low anterior resection; MIRECA, minimally invasive rectal cancer treatment Taskforce; MRI, magnetic resonance imaging; n, number of patients; TEM, transanal endoscopic microsurgery; TME, total mesorectal excision.

A total of 2773 patients were initially screened for eligibility, with exclusions made following the criteria outlined in the Materials and Methods Section (Figure 2). Ultimately, 487 patients across eight participating centers were included in the final analysis. Interobserver reliability for pelvic measurements ranged from good to excellent, with an intraclass correlation coefficient (ICC) exceeding 0.75 across all parameters.

### 3.2. The Relationship Between the Preoperative Factors and AL

Detailed patient and tumor characteristics, including pelvimetric measurements, are presented in Table 1. The mean age of patients at the time of surgery was 66 ± 9 years, with 298 (61.2%) being male. Diverting protective stomas were created in 239 patients (49%), and AL occurred in 70 patients (14%). Three patients showed AL grade A, 21 grade B, and 46 grade C. Based on univariate analysis, several factors were identified to be associated with AL (Table 2). These included male sex, distance to the ARJ, clinical tumor staging, pelvic inlet, interspinous distance, and pelvic volume. The individual correlations of the significant relationships were all below 0.2, indicating moderate individual predictive value. The multivariate regression analysis identified distance to the ARJ, pelvic inlet, and interspinous distance as independent predictors for AL. The variance inflation factor (VIF) of the independent variables was below two.

### 3.3. Performance of Machine Learning Models

**Figure 3:**
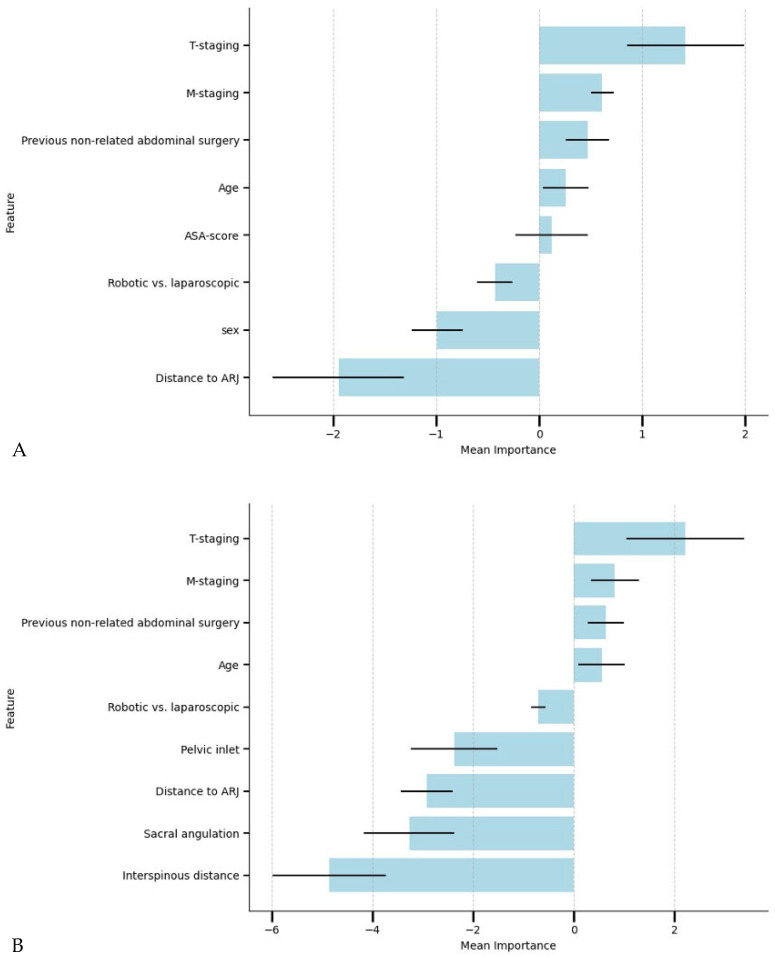
The variables and their importance used by the best-performing models (Model (**A**): only clinical characteristics, Model (**B**): clinical characteristics and pelvimetry). The mean value and standard deviation are shown for the importance based on the five-fold cross-validation of the models. Abbreviations: T-staging, clinical tumor staging, M-staging, clinical metastasis staging.

Table 3 shows the models’ performances for predicting AL. The LR models had the highest F1 score. The LR model including pelvimetric dimensions obtained the highest AUC-ROC and AUC-PR. For all three ML model types, the models with pelvimetry generated a higher ROC-AUC than those developed without pelvimetry. The variables used by the best-performing models with and without pelvimetry (Models A and B) and their individual importance are shown in Figure 3. Model A incorporated the clinical and pelvimetric predictors: clinical tumor staging, clinical metastasis staging, previous non-related abdominal surgery, age, surgical approach, pelvic inlet, distance to ARJ, sacral angulation, and interspinous distance. Model B incorporated only the identified independent clinical predictors: clinical tumor staging, clinical metastasis staging, previous non-related abdominal surgery, age, ASA-score, surgical approach, sex, and distance to ARJ. Model A used borderline SMOTE, and Model B used a 1:5 weighting for compensation of data imbalance. While the AUC-ROC and AUC-PR values for Model A—including pelvimetric measurements—were higher than those for Model B, the difference did not reach statistical significance (*p* > 0.05).

## 4. Discussion

This multicenter retrospective study of 487 MRI-defined rectal cancer patients provides new insights into the role of pelvic anatomy in predicting AL following restorative rectal cancer surgery. This study demonstrated that specific pelvic measurements, such as distance to ARJ, pelvic inlet width, and interspinous distance, were independently associated with AL risk. Incorporating pelvimetric measurements into ML models improved predictive performance compared to models based solely on clinical variables. While models including pelvimetric measurements yielded higher AUC-ROC and AUC-PR values, the improvement was not statistically significant. These results suggest that pelvic measurements hold promise for refining preoperative risk stratification, but further research is needed to improve model accuracy and clinical applicability.

The results of this study add to a growing investigation of pelvic anatomy as a potential determinant of surgical outcomes in rectal cancer surgery. The observed inverse relationship between pelvimetric dimensions and AL suggests that anatomical constraints within the pelvis may exacerbate technical challenges, particularly in achieving adequate visualization and constructing tension-free anastomoses. Specifically, a narrower pelvic inlet may limit the surgeon’s ability to maneuver instruments effectively, increasing the difficulty of precise dissection and anastomotic construction. Likewise, a reduced interspinous distance may contribute to restricted access to the distal rectum, potentially increasing anastomotic tension and impairing vascular perfusion, both of which are known contributors to AL. However, while these anatomical factors are mechanistically plausible contributors to AL, evidence supporting their direct role remains inconclusive. Prior studies have yielded conflicting results regarding the specific pelvic dimensions associated with AL, reflecting the multifactorial nature of AL risk. For instance, Yu et al. identified pelvic inlet width as an independent predictor of AL [24], while Atasoy et al. [25] found no such association but highlighted the pelvic depth as a significant predictor. Differences in measurement protocols, study populations, or interactions between pelvic anatomy and other risk factors such as tumor characteristics, perioperative management, and surgical expertise may explain these discrepancies. Moreover, while this study focused on preoperative anatomical factors, additional variables such as surgical technique, patient inflammatory status, and markers of tissue healing could further enhance predictive models. Intraoperative factors, including anastomotic perfusion assessment and surgical approach, may also play a significant role in refining risk estimation. Furthermore, most studies have focused on surrogate markers of surgical difficulty, (e.g., prolonged operative time or incomplete mesorectal excision), rather than directly linking pelvimetric anatomy to AL risk [7,8,9,10,26]. This underscores the need for further research designed specifically to evaluate the predictive value of pelvic measurements in the context of AL, while also incorporating a broader range of clinical and biological factors that may influence anastomotic integrity.

The current analysis demonstrated moderate predictive performance across all ML models, with the logistic regression model incorporating pelvimetry achieving the highest AUC-ROC of 0.70. While an AUC-ROC of 0.70 is insufficient to guide intraoperative decision-making or dictate surgical strategy reliably, it may still provide value in the preoperative setting. Specifically, such models can support shared decision-making by helping surgeons discuss individual AL risk with patients, allowing for more informed conversations about potential postoperative complications and the need for protective measures such as a defunctioning stoma. Although this performance is relatively modest, it underscores the complexity of AL prediction, which is influenced by a range of clinical, anatomical, and intraoperative factors. To reduce potential intra-operative variability, we limited the cohort to elective laparoscopic and robotic TME procedures at high-volume centers. While this approach helped control for surgeon experience and procedural consistency, it also reduced generalizability. Compared with existing AL prediction models incorporating a broader set of clinical and intraoperative variables, our model demonstrated similar or slightly lower discriminative performance [16,27,28]. These findings highlight the challenge of accurately predicting AL based solely on preoperative factors and suggest that integrating additional perioperative and intraoperative predictors may enhance predictive performance. Notably, while several variables were statistically significant in univariate analysis, their correlation values were below 0.2, indicating weak individual associations with AL risk. This suggests that no single preoperative factor is strongly predictive in isolation, but instead that a combination of anatomical and clinical variables may contribute to overall risk stratification. Future predictive models should incorporate larger datasets, additional clinical and surgical parameters, and intraoperative variables to improve accuracy and broaden applicability. Further research should assess the clinical relevance of these predictors by evaluating effect sizes and their impact on surgical decision-making. Based on a more extensive dataset, the ML methods will be better able to find a non-linear relationship between patient characteristics and AL. The ability of ML to recognize complex patterns and thereby improve prediction models has already been shown for cancer prognosis and recurrence [29]. In addition, future work could delve into the use of deep learning methods.

Integrating pelvic measurements into preoperative predictive models may offer several advantages, particularly in providing a more individualized risk assessment. Feature importance analysis in our ML models (Figure 3) revealed that pelvic dimensions were among the most influential predictors of AL, surpassing several traditional clinical variables. Interestingly, feature importance analysis identified sex as a strong and significant predictor in models without pelvimetric parameters, while sex was not a significant predictor when pelvic measurements were included, likely due to collinearity. This finding highlights the limitations of using sex as a surrogate for pelvic anatomy. For instance, male patients with relatively large pelvic dimensions may be misclassified as high-risk when sex alone is used as a predictor. Pelvimetric measurements, in contrast, offer a more personalized risk profile, capturing variability within both sexes and providing a more objective assessment of anatomical challenges. Acknowledging well-established physiological differences in pelvic anatomy between males and females, the current study was underpowered to develop separate sex-specific models. However, future research should explore whether sex-specific models improve predictive accuracy and further investigate the interaction between sexes and pelvic dimensions. Such analyses could help refine risk stratification and optimize surgical decision-making by accounting for anatomical variability between sexes.

Automating the assessment of pelvic dimensions through MRI-based algorithms could further enhance their clinical utility [30]. Despite emerging studies reporting a potential association between pelvic anatomy and surgical outcomes, pelvimetric measurements are not widely used for AL prediction due to several challenges. Manual measurements are time-consuming, require specialized imaging expertise, and are subject to interobserver variability, limiting their adoption in clinical practice. Additionally, standardized measurement protocols are lacking, leading to inconsistencies across studies and clinical settings. Automated pelvimetric tools could overcome these barriers by providing real-time, objective, and reproducible risk assessments, offering surgeons objective insights into anatomical constraints and aiding preoperative planning. Although the inclusion of pelvic measurements did not result in a statistically significant improvement in model performance (*p* > 0.05), their clinical utility should not be overlooked. Preoperative awareness of pelvic anatomy can help surgeons anticipate technical challenges and optimize surgical planning. By quantifying individual risk, surgeons may be better equipped to balance the need for protective measures against the risks associated with unnecessary stomas. Avoiding stomas in low-risk patients may contribute to improved quality of life [31,32], a reduction in stoma-related complications [33], shorter hospital stays, and overall cost savings. Conversely, high-risk patients identified through predictive modeling may benefit from early diversion, potentially mitigating the morbidity associated with AL. A prospective study to validate the integration of automated risk profiles into routine preoperative assessment may demonstrate their utility in reducing postoperative complications and improving patient outcomes.

This study has several limitations that warrant consideration. First, the retrospective design introduces the potential for selection bias, and the cohort—drawn exclusively from high-volume centers—may not represent outcomes in less specialized settings. Additionally, although efforts were made to standardize protocols and train participating radiologists, interobserver variability in pelvimetric measurements remains a concern. Finally, the sample size, with only 70 AL events, may have limited the models’ discriminatory power. Future research should prioritize prospective multicenter designs with larger sample sizes and expanded predictor variables to improve model accuracy and external validity.

## 5. Conclusions

In conclusion, this multicenter retrospective study provides new insights into the role of pelvic anatomy, as assessed through MRI-based pelvimetry, in predicting AL following restorative rectal cancer surgery. While previous studies have suggested an association between pelvic dimensions and surgical difficulty, our findings demonstrate that the pelvic inlet and interspinous distance were independently associated with AL and influenced predictive modeling. Although these models are not yet sufficiently accurate to guide intraoperative decision-making, they may serve as an additional preoperative risk assessment tool, supporting surgeons in identifying higher-risk patients and facilitating informed discussions with patients. Future prospective studies with larger datasets and standardized protocols are essential to validate and refine these models, paving the way for more clinically applicable risk stratification tools that enhance patient outcomes in rectal cancer surgery.

## Figures and Tables

**Table 1:** The relationship between clinical characteristics and anastomotic leakage. Abbreviations: AL, anastomotic leakage; ARJ, anorectal junction; ASA, American Society of Anesthesiologists; BMI, body mass index; c, coefficient; n, number of patients. Significant variables in univariate analyses were underlined.

Variable	Overall (n = 487)	No AL (n = 417)	AL (n = 70)	Univariate			Multivariate
				*p*	c	OR [CI]	*p*
Patient and clinical factors							
Sex (%)				0.002	0.140	0.38 [0.21–0.70]	
Male	298 (61.2)	243 (58.3)	55 (78.6)				
Female	189 (38.8)	174 (41.7)	15 (21.4)				
Age (year)	65.6 [12.0]	65.3 [12.0]	67.0 [12.7]	0.144	0.066		
Height (cm)	174.3 [13.0]	174.1 [13.0]	175.7 [12.0]	0.140	0.067		
Weight (kg)	79.7 [21.0]	79.5 [22.0]	80.9 [15.5]	0.438	0.035		
BMI (kg/m^2^)	26.2 [5.2]	26.2 [5.3]	26.1 [4.7]	0.992	−0.000		
ASA-score				0.864	0.039		
1	111 (22.8)	97 (23.3)	14 (20.0)				
2	300 (61.6)	254 (60.9)	46 (65.7)				
3	75 (15.4)	65 (15.6)	10 (14.3)				
4	1 (0.2)	1 (0.2)	0 (0.0)				
Previous non-related abdominal surgery	124 (25.5)	104 (24.9)	20 (28.6)	0.619	0.023		
Tumor factors							
T-stage				0.037	0.132	1.79 [1.16–2.76]	
1	21 (4.3)	21 (5.0)	0 (0.0)				
2	173 (35.5)	155 (37.2)	18 (25.7)				
3	274 (56.3)	225 (54.0)	49 (70.0)				
4	19 (3.9)	16 (3.8)	3 (4.3)				
N-stage				0.384	0.063		
0	243 (49.9)	213 (51.1)	30 (42.9)				
1	156 (32.0)	129 (30.9)	27 (38.6)				
2	88 (18.1)	75 (18.0)	13 (18.6)				
M-stage				0.115	0.072		
0	466 (95.7)	402 (96.4)	64 (91.4)				
1	21 (4.3)	15 (3.6)	6 (8.6)				
Distance to ARJ (cm)	8.3 [4.0]	8.6 [4.5]	7.0 [4.0]	0.000	−0.173		<0.001
Treatment factors							
Radiotherapy				0.132	0.107		
None	247 (50.7)	219 (52.5)	28 (40.0)				
Short	140 (28.7)	112 (26.9)	28 (40.0)				
Long	5 (1.0)	4 (1.0)	1 (1.4)				
Chemoradiation	95 (19.5)	82 (19.7)	13 (18.6)				
Surgical approach				0.084	0.078		
Robotic	299 (61.4)	249 (59.7)	50 (71.4)				
Laparoscopic	188 (38.6)	168 (40.3)	20 (28.6)				

**Table 2:** The relationship between pelvimetry and anastomotic leakage. Abbreviations: AL, anastomotic leakage; c, coefficient; n, number of patients. Significant variables in univariate analyses were underlined.

Variable	Overall (n = 487)	No AL (n = 417)	AL (n = 70)	Univariate		Multivariate
				*p*	c	*p*
Sacrococcygeal depth	127.1 ± 13.5	127.3 ± 13.5	125.6 ± 13.4	0.318	−0.045	
Pelvic inlet	114.5 ± 11.0	115.2 ± 11.2	110.5 ± 9.1	0.001	−0.152	0.004
Pelvic outlet	87.7 ± 8.8	87.9 ± 8.8	87.0 ± 8.8	0.463	−0.033	
Pelvic depth	157.5 ± 15.4	157.5 ± 15.6	157.4 ± 14.4	0.949	−0.003	
Intertuberous distance	101.0 [20.7]	101.4 [20.0]	98.9 [20.1]	0.119	−0.071	
Interspinous distance	105.7 [15.8]	106.5 [15.4]	101.1 [13.9]	0.000	−0.165	0.013
Sacral angulation	111.6 [13.9]	111.9 [14.5]	109.7 [11.4]	0.076	−0.080	
Pelvic area (A–E)	225.7 ± 26.8	226.4 ± 26.9	221.7 ± 25.9	0.175	−0.062	
Pelvic volume	687.5 [201.4]	694.1 [211.6]	647.6 [151.5]	0.014	−0.111	

**Table 3:** Performance of the three machine learning models for predicting anastomotic leakage developed with and without pelvimetry. The mean and standard deviation of the five-fold cross-validation are provided. The values of the best-performing models are highlighted in bold. Abbreviations: AUC, area under the curve; LR, logistic regression; PR, precision–recall; RFC, random forest classifier; ROC, receiver operating characteristic; XGBoost, Extreme Gradient Boosting.

Model		F1-Score	ROC-PR	ROC-AUC	Sensitivity	Specificity
LR	+pelvimetry	**0.34 ± 0.06**	**0.32 ± 0.10**	**0.70 ± 0.09**	**0.64 ± 0.29**	0.65 ± 0.11
	−pelvimetry	0.34± 0.07	0.27± 0.08	0.68± 0.04	0.57± 0.07	0.70 ±0.08
RFC	+pelvimetry	0.33 ± 0.12	0.25 ± 0.08	0.67 ± 0.12	0.51 ± 0.13	**0.72 ± 0.15**
	−pelvimetry	0.29± 0.06	0.25± 0.09	0.63± 0.05	0.64± 0.29	0.55 ± 0.16
XGBoost	+pelvimetry	0.30 ± 0.08	0.22 ± 0.07	0.63 ± 0.11	0.57 ± 0.36	0.65 ± 0.25
	−pelvimetry	0.28 ± 0.10	0.22 ± 0.08	0.61 ± 0.07	0.47 ± 0.26	0.69 ± 0.12

## Data Availability

The data presented in this study may be made available on reasonable request and reviewing by the MIRECA Taskforce reviewing committee.

## Abbreviations

The following abbreviations are used in this manuscript:

MDPI	Multidisciplinary Digital Publishing Institute
DOAJ	Directory of open access journals
TLA	Three-letter acronym
LD	Linear dichroism
TME	Total Mesorectal Excision
AL	Anastomotic Leakage
ML	Machine Learning
MIRECA	Minimally Invasive Rectal Cancer Taskforce
RLAR	Restorative Low Anterior Resection
MRI	Magnetic Resonance Imaging
DCRA	Dutch ColoRectal Audit
ARJ	Anorectal Junction
TEM	Transanal Endoscopic Microsurgery
NRLAR	Non-Restorative Low Anterior Resection
APR	Abdominoperineal Resection
STROBE	Strengthening the Reporting of Observational Studies in Epidemiology
MEC-U	Medical Research Ethics Committees United
ISREC	International Study Group of Rectal Cancer
T2	T2-Weighted Imaging (MRI Sequence)
ICC	Intraclass Correlation Coefficient
SD	Standard Deviation
IQR	Interquartile Range
MAR	Missing at Random
MICE	Multiple Imputation by Chained Equations
ASA	American Society of Anesthesiologists
LR	Logistic Regression
RFC	Random Forest Classifier
XGB	XGBoost
SMOTE	Synthetic Minority Oversampling Technique
AUC-PR	Area Under the Precision-Recall Curve
AUC-ROC	Area Under the Receiver Operating Characteristic Curve
